# Pharmacogenetics in the Treatment of Huntington’s Disease: Review and Future Perspectives

**DOI:** 10.3390/jpm13030385

**Published:** 2023-02-22

**Authors:** Xandra García-González, Esther Cubo, Lucía Simón-Vicente, Natividad Mariscal, Raquel Alcaraz, Laura Aguado, Jéssica Rivadeneyra-Posadas, Antonio Sanz-Solas, Miriam Saiz-Rodríguez

**Affiliations:** 1 Pharmacy Department, Instituto de Investigación Sanitaria Gregorio Marañón (IiSGM), Hospital General Universitario Gregorio Marañón, 28007 Madrid, Spain; 2 Neurology Department, Hospital Universitario de Burgos, 09006 Burgos, Spain; 3 Department of Health Sciences, University of Burgos, 09001 Burgos, Spain; 4 Research Unit, Fundación Burgos por la Investigación de la Salud (FBIS), Hospital Universitario de Burgos, 09006 Burgos, Spain

**Keywords:** Huntington, pharmacogenetics, antichoreic, antidepressant, antipsychotic

## Abstract

Huntington’s disease (HD) is an autosomal dominant progressive brain disorder, caused by a pathological expansion of a CAG repeat that encodes the huntingtin gene. This genetic neurodegenerative rare disease is characterized by cognitive, motor, and neuropsychiatric manifestations. The aim of the treatment is symptomatic and addresses the hyperkinetic disorders (chorea, dystonia, myoclonus, tics, etc.) and the behavioural and cognitive disturbances (depression, anxiety, psychosis, etc.) associated with the disease. HD is still a complex condition in need of innovative and efficient treatment. The long-term goal of pharmacogenetic studies is to use genotype data to predict the effective treatment response to a specific drug and, in turn, prevent potential undesirable effects of its administration. Chorea, depression, and psychotic symptoms have a substantial impact on HD patients’ quality of life and could be better controlled with the help of pharmacogenetic knowledge. We aimed to carry out a review of the available publications and evidence related to the pharmacogenetics of HD, with the objective of compiling all information that may be useful in optimizing drug administration. The impact of pharmacogenetic information on the response to antidepressants and antipsychotics is well documented in psychiatric patients, but this approach has not been investigated in HD patients. Future research should address several issues to ensure that pharmacogenetic clinical use is appropriately supported, feasible, and applicable.

## 1. Introduction

Rare neurodegenerative diseases are fatal, with no available therapy to cure or slow down their progression. Over the last decades, there has been a growing interest in research on rare diseases, often supported by regulatory agencies. Patients and health professionals showed different research priorities for rare neurological diseases. Interestingly, patient representatives indicated that therapies are of the utmost importance since their lives are often heavily impacted, and their main goal is to relieve the burden of disease [1]. Among rare diseases, Huntington’s disease (HD) is of special interest given the easy access to diagnosis in developed countries. HD is an autosomal dominant progressive brain disorder, caused by a pathological expansion of a CAG repeat (≥36 repeats) that encodes the huntingtin gene (HTT). This genetic neurodegenerative disease is characterized by cognitive, motor, and neuropsychiatric manifestations. The worldwide prevalence of HD is 2.7 per 100,000 [2]. Every attempt to prevent or slow HD progression in patients and mutation carriers has failed so far [3].

Clinical manifestations of HD are associated with the loss of neurons, especially in the cortex and striatum, caused by the expansion of the huntingtin protein. This affects neurotransmission mediated by dopamine and glutamate, which is why these neurotransmitters are currently the main target of available pharmacotherapy [4]. The aim of the treatment is symptomatic and addresses the hyperkinetic disorders (chorea, dystonia, myoclonus, tics, etc.) and the behavioural and cognitive disturbances (depression, anxiety, psychosis, etc.) associated with the disease.

Tetrabenazine and deutetrabenazine (central monoamine depletors) are indicated for the treatment of chorea, whereas neuroleptics are commonly used for the treatment of chorea and psychosis related to HD. In addition, since HD is a multisymptomatic disease, patients often receive other concomitant medication to treat symptoms such as depression, irritability, apathy, or anxiety.

Variations in response to drugs may be pharmacodynamic or pharmacokinetic and, therefore, affect the efficacy and toxicity of drugs. Response rates to medications, used to treat a wide range of disorders, typically range from 50% to 75%, meaning that up to half of patients see no benefit [5]. In addition, many individuals suffer from adverse drug reactions (ADRs). Nearly 8.4% of hospital admissions in Europe are related to ADRs, and about 10% of patients suffer an ADR during hospitalization [6,7]. In the USA, it is estimated that severe drug toxicities cause over 100,000 deaths and cost USD 30–100 billion annually [8]. Inter-individual variability in drug response affects not only patient well-being, but also poses enormous clinical and financial burdens. It is well known that antidopaminergic drugs improve chorea but, on the other hand, can induce significant morbidity and mortality, including depression with suicidal outcomes, obesity, diabetes, metabolic syndrome, and cardiotoxicity in HD [9]. According to preclinical and clinical studies, the side effects of anticholinergic drugs can be attributed to the drugs’ multiple binding to dopamine, histamine, 5-hydroxytryptiamine, histamine acetylcholine, adrenergic, NMDA, and GABAa receptors. The efficacy and ADR profiles of antichoreic drugs are heterogeneous with large interindividual variability. As a result, treatment selection remain a largely trial-and-error process in HD. In this regard, there is a growing interest on pharmacogenetics, because it could provide important information about genetic variants in the antichoreic metabolizing enzymes and receptors, and, therefore, help clinicians implement personalized medicine. The aim of this study is to carry out a review of the available publications and evidence related to the pharmacogenetics of HD, with the objective of compiling all information that may be useful in optimizing drug therapy in these patients.

## 2. Materials and Methods

This systematic review was conducted according to the preferred reporting items for systematic reviews and meta-analyses (PRISMA) statements [10]. The literature search included the following databases: MEDLINE, PharmGKB [11], EU Clinical Trials Register and ClinicalTrials.gov. The search strategy included similar keywords in all databases: Huntington, Huntington’s disease, polymorphism (or SNP, or single nucleotide polymorphism or pharmacogenetics or pharmacogenomics); and the drugs included in the International Guidelines for the Treatment of Huntington’s Disease [12]: monoamine transporter type 2 (VMAT2) inhibitors (tetrabenazine, deutetrabenazine), first-generation antipsychotic drugs (haloperidol), second-generation antipsychotic drugs (aripiprazole, olanzapine, clozapine, risperidone), selective serotonin reuptake inhibitors (citalopram, escitalopram, fluoxetine, paroxetine, sertraline), and benzodiazepines (diazepam, etizolam, quazepam, desmethylclobazam, alprazolam, midazolam, clonazepam, lorazepam). Two independent reviewers (XGG and MSR) individually screened all the titles and abstracts and evaluated each article. Disagreements were resolved by consensus and in concordance with a third reviewer (EC), when necessary. Studies that fulfilled the following criteria were included: (1) studies with pharmacogenetic biomarkers analysed in diagnosed HD patients’ cohorts, in which any of the following were prescribed: tetrabenazine, deutetrabenazine, haloperidol, chlorpromazine, pluphenazine, aripiprazole, olanzapine, quetiapine, risperidone, citalopram, escitalopram, fluoxetine, paroxetine, sertraline, amitriptyline, desipramine, doxepin, imipramine, nortriptyline, trimipramine, diazepam, etizolam, quazepam, desmethylclobazam, alprazolam, midazolam, clonazepam, lorazepam, and (2) studies reporting efficacy and outcome variables. The European Medicines Agency (EMA) and U.S. Food and Drug Administration (FDA) drug labels were reviewed in search of any recommendations based on patient genotype. Additionally, the most recent guidelines and publications by the Clinical Pharmacogenetics Implementation Consortium (CPIC) and Dutch Pharmacogenetics Working Group (DPWG) were also reviewed for any drug-gene recommendations.

## 3. Pharmacogenetics of the Drugs Used in the Management of Chorea in Huntington’s Disease

**Figure 1:**
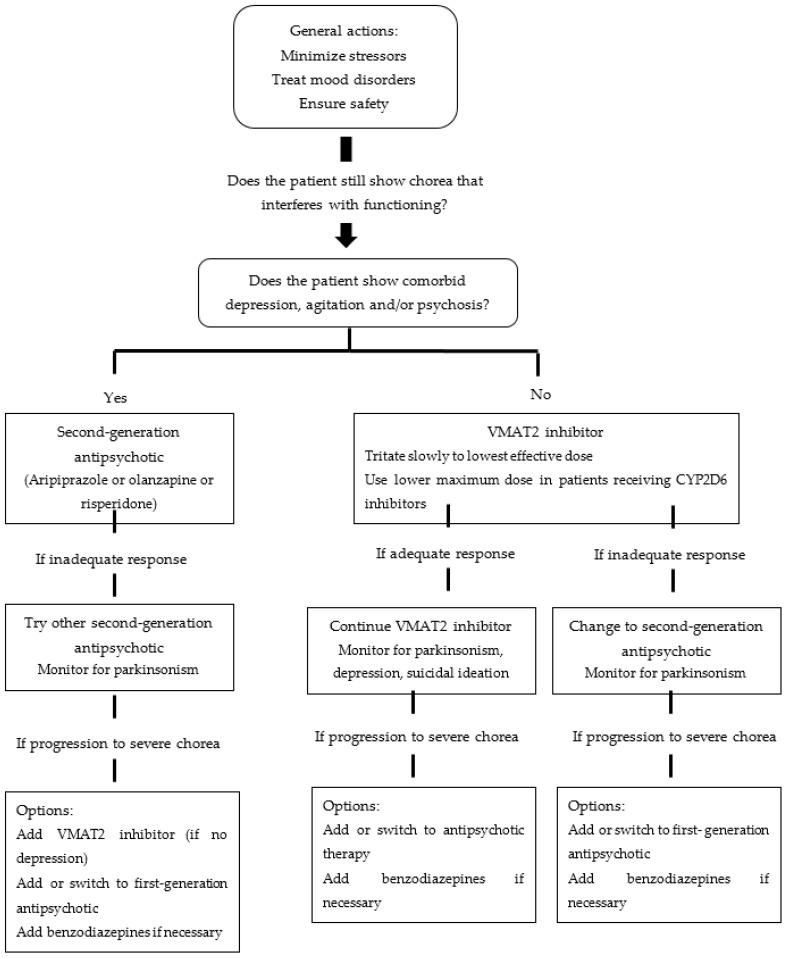
Treatment algorithm for chorea in HD, adapted from Suchowersky et al.’s *Huntington Disease: Management* [13].

The pharmacologic treatment of patients suffering from chorea is indicated only in those cases in which this symptom interferes with the patient’s functionality [4]. Controlling this symptom can reduce the risk of falls or choking and improve the patient´s speech and rest, but the medications used can induce parkinsonism, depression and suicidal thoughts. For this reason, the decision to start treatment must always be based on a comprehensive risk–benefit analysis. This article will review the pharmacogenetic data of the drugs used according to the treatment algorithm for chorea in HD (Figure 1) [13].

### 3.1. Monoamine Transporter Type 2 (VMAT2) Inhibitors (Tetrabenazine and Deutetrabenazine)

Vesicular monoamine transporter type 2 (VMAT2) inhibitors are usually considered the drugs of choice for the management of chorea, except for patients with depression, due to the risk of worsening depression and suicidality.

Tetrabenazine is the originally commercialized molecule, and in 2017 the FDA approved Deutetrabenazine (Austedo^®^), which is an isotopic isomer of tetrabenazine. By incorporating deuterium instead of hydrogen in six positions, the molecule shows slower metabolism, which allows for fewer daily administrations [14]. Deutetrabenazine is currently not commercialized in Europe and other countries.

Drowsiness, sedation, extrapyramidal symptoms, depression, akathisia, and parkinsonism are common side effects of VMAT2 inhibitors. There is also an increased risk of suicidality, QT prolongation, and neuroleptic malignant syndrome [15,16].

Both tetrabenazine and deutetrabenazine are rapidly transformed by carbonyl reductase to their respective nondeuterated and deuterated metabolites, α-dihydrotetrabenazine (α-HTBZ) and β-dihydrotetrabenazine (β-HTBZ), that bind selectively to VMAT2, thus inhibiting monoamine transport to the interior of presynaptic neuronal vesicles, effectively depleting dopamine and other monoamines in the central nervous system.

α-HTBZ and β-HTBZ are mainly metabolized by cytochrome P450 (CYP) 2D6, with minor contributions from CYP1A2 and CYP3A4/5 [17].

CYP2D6 is an enzyme that participates in the metabolism of around a quarter of the drugs used in therapeutics [18]. The *CYP2D6* gene is highly polymorphic with genetic variants that have substantial functional consequences, including reduced (e.g.,*9,*10,*17,*41) and non-functional alleles (e.g., *3,*4,*5,*6), gene deletions, and duplications [19]. Based on their capacity to metabolize CYP2D6 substrates, patients can be categorized as ultrarapid metabolizers (UM), normal metabolizers (NM), intermediate metabolizers (IM), and poor metabolizers (PM) [20]. Patient metabolic ability can also be affected by the concomitant administration of strong enzyme inhibitors, such as paroxetine, fluoxetine, quinidine, or bupropion [21].

Patients with impaired CYP2D6 metabolism have higher plasma concentrations of its substrates due to accumulation, and this can lead to variations in response and/or a higher risk of drug-induced adverse events.

The recommended initial dose of tetrabenazine is 25 mg three times a day, and it can be increased every 3 or 4 days, at a rate of 25 mg/day, up to a maximum of 200 mg/day, or if the tolerance limit is reached due to undesirable effects. *CYP2D6* genotyping is required by the FDA and the Swiss Agency for Therapeutic Products (Swissmedic). Both drug labels state that patients requiring doses above 50 mg per day of tetrabenazine should be genotyped due to the increased risk for adverse events in patients with impaired metabolism. The US drug label also states that patients with a CYP2D6 PM phenotype should not exceed a total daily dose of 50 mg, with a maximum of 25 mg administered per dose. The recommended dose for CYP2D6 IM and NM is 100 mg, with a maximum single dose of 37.5 mg.

The European Summary of Product Characteristics only states that tetrabenazine metabolites are substrates of CYP2D6 and that Dosing, therefore, may be influenced by the patient´s CYP2D6 metabolic status and the concomitant administration of CYP2D6 potent inhibitors [22].

Only one study has evaluated the clinical implications of CYP2D6 pharmacogenetic profiling in patients treated with tetrabenazine [23]. *CYP2D6* genotyping was performed in 127 patients treated with tetrabenazine, and the duration of titration to a stable dose, total daily dose, response rating scores, and adverse events were retrospectively analysed. Titration time was significantly longer in UM than in NM, IM and PM (8 weeks vs. 3.3, 4.4, and 3 respectively *p* < 0.01) and average stable daily doses were higher. When compared to NM, IM had a worse clinical response (*p* = 0.013). No statistically significant differences in the incidence of adverse events were detected according to metabolic status. These findings led the authors to doubt the advice on *CYP2D6* genotyping that is currently provided.

Deutetrabenazine is indicated for the treatment of chorea associated with Huntington’s disease and tardive dyskinesia in the US. The recommended starting dose is 6 mg once daily, and it can be up-titrated at weekly intervals by 6 mg per day, up to a maximum recommended daily dosage of 48 mg (24 mg twice daily). The FDA drug label states that in patients carrying the CYP2D6 PM phenotype or in patients receiving strong CYP2D6 inhibitors (e.g., quinidine, antidepressants such as paroxetine, fluoxetine, and bupropion), the total daily dosage of deutetrabenazine should not exceed 36 mg (maximum single dose of 18 mg).

No clinical consensus guidelines are currently available for genotyping of *CYP2D6* and dose adjustment of tetrabenazine and deutetrabenazine.

### 3.2. Second-Generation Antipsychotic Drugs (Aripiprazole, Olanzapine, and Risperidone)

Antipsychotics are used for the treatment of chorea in those patients for whom VMAT2 inhibitors are contraindicated (e.g., depression) or when they fail to control symptoms and can also help with HD psychiatric symptoms [24,25,26,27]. Second generation antipsychotics act by blocking serotonin receptors, especially 5-HT2A, as well as D2 dopamine receptors, and are usually preferred to first-generation drugs, which are discussed in the next paragraph, because of their lower risk of extrapyramidal effects.

CYP2D6 is the main enzyme involved in the metabolism of aripiprazole and risperidone, although other CYP450 enzymes, such as CYP3A4, also play a role [28,29]. Available evidence and current pharmacogenetic recommendations for these drugs are mainly related to CYP2D6 metabolizer status, although studies have been carried out in psychiatric populations and not specifically in HD patients.

Although there is substantial evidence linking aripiprazole and its active metabolite, dehydroaripiprazole, to the CYP2D6 phenotype, no direct impact on clinical outcomes has been established [30,31,32,33]. Hence, dosing recommendations are based mainly on available pharmacokinetic studies.

Both the American and European drug agencies consider that dose adjustments are necessary for CYP2D6 PMs taking aripiprazole. A 50% dose reduction is recommended for the oral presentations, and the starting and maintenance dose of the intramuscular prolonged release injectable should be 300 mg (instead of 400 mg) in known CYP2D6 PM, and this should be further reduced to 200 mg if strong CYP3A4 inhibitors are concomitantly used [34,35]. The DPWG also recommends reducing the maximum dose of aripiprazole to 68–75% for patients carrying CYP2D6 PM phenotypes [36]. The DPWG suggests that genotyping patients before starting aripiprazole may be helpful in preventing adverse outcomes. Genotyping can be considered on an individual patient basis. However, the DPWG advises following the gene-drug guideline if the genotype is available [36]. Variants in *DRD2*, *ANKK1* and *DAOA* genes have been associated with aripiprazole efficacy in schizophrenic patients [37,38,39,40]. However, the evidence is still insufficient to provide specific recommendations.

Strong evidence (level 1A) links the *CYP2D6* genotype, and risperidone clearance and plasma concentrations [41,42]. As in the case of aripiprazole, dose adjustment recommendations are mainly based on this established pharmacokinetic relationship [36].

In their 2020 guideline update, the DPWG categorized *CYP2D6* genotyping as potentially beneficial before the initiation of risperidone treatment, for the prevention of side effects and for drug effectiveness [43]. When genotype is available, it is recommended to reduce the dose for CYP2D6 PM and use an alternative drug, or to titrate the dose according to the maximum dose for the active metabolite for CYP2D6 UM [43].

Variants in several pharmacogenes, including *ABCB1, AKT1, CCL2* and *COMT*, have been associated with the risperidone response, but no strong relationship has been established yet [44,45,46,47].

Glucuronidation is the main metabolic pathway for olanzapine, but CYP1A2, CYP2D6 and CYP3A4 also play a role [48]. No recommendations on drug labels and/or pharmacogenetic guidelines are available for olanzapine. However, patients using fluvoxamine or other CYP1A2 inhibitors should be given olanzapine at a reduced starting dose, considering CYP1A2 is highly involved in its metabolism. A recent systematic review showed that multiple studies found strong evidence linking *DRD2* Taq1A (rs1800497) *A1, *LEP*-2548 (rs7799039) G and CYP1A2*1F alleles to variations in the efficacy and safety of olanzapine. With a reasonable degree of evidence, *DRD2*-141 (rs1799732) Ins, A-241G (rs1799978) G, *DRD3* Ser9Gly (rs6280) Gly, *HTR2A* rs7997012 A, *ABCB1* C3435T (rs1045642) T and G2677T/A (rs2032582) T and UGT1A4*3 alleles were related to safety, efficacy, and/or pharmacokinetic variability [49]. Moreover, carriers of *UGT1A4* 142T > G were shown to have a decrease in daily dose-corrected plasma concentrations of 25% in schizophrenic patients [50].

Relating to adverse events, polymorphisms in *DRD2* have been associated with antipsychotic-related hyperprolactinemia or weight gain [51,52]. Cannabinoid receptor 1 (*CNR1*) and leptin gene (LEP) may also be associated with weight gain in schizophrenia patients treated with first and second generation antipsychotics [53,54]. The study performed by Koller et al. described that short-term treatment with aripiprazole and olanzapine had a significant influence on metabolic parameters, such as prolactin levels, higher C-peptide, glucose and insulin levels, among others, and that these were related to polymorphisms in *DRD3, CYP3A, COMT, UGT1A1, APOC3* and *HTR2A* [55]. Once more, there is a lack of studies performed on HD patients.

### 3.3. First-Generation Antipsychotic Drugs (Haloperidol)

First-generation antipsychotics are relegated to those cases of severe chorea unresponsive to VMAT inhibitors and second-generation antipsychotics, due to their higher potency. However, they have a higher risk of side effects, such as sedation, dystonia, parkinsonism, hypotension, or akathisia.

Haloperidol is probably the most commonly used option, in doses from 0.5 mg to 10 mg per day [56]. It is mainly glucoronized (50–60%) by UGT2B7, UGT1A9, and UGT1A4, but around 25% of the administered dose undergoes reduction by CYP3A4 (main enzyme) and CYP2D6 [57,58]. Despite its secondary role, several studies have proven the relationship between CYP2D6 metabolizer status and haloperidol plasma levels [59,60,61].

For this reason, the DPWG recommends using 60% of the standard dose of haloperidol for CYP2D6 PM and 1.5 times the standard dose for CYP2D6 UM, or selecting an alternative drug [62].

### 3.4. Other Drugs Used in the Management of Chorea

Benzodiazepines, such as clonazepam and lorazepam, may be used in the short term to decrease severe episodes of chorea, but their use is not recommended in the long term. The polymorphic CYP2C19 and CYP3A4/5 enzymes primarily or partially metabolize a number of benzodiazepines. *CYP2C19* polymorphism has been shown to affect the pharmacokinetics of diazepam, etizolam, quazepam, and desmethylclobazam [63]. Diazepam is metabolized by CYP2C19 and CYP3A4. A dose reduction of 50% and 20–30% may be adequate for CYP2C19 PM and IM, respectively [64]. On the contrary, UM may benefit from a dose increase of 25–50% [64]. The metabolism of clobazam involves CYP2C19 and CYP3A4. The drug label for clobazam includes dosage modifications based on the phenotype of CYP2C19: PMs should start with a dose of 5 mg/day and titrate the dose gradually based on weight, with a maximum dose of half the recommended dose [64]. Significant CYP3A4 metabolism occurs with midazolam, clonazepam, alprazolam and triazolam. There are no published guidelines at this time since little research has been done on the potential effects of the CYP3A4 phenotype on exposure and safety [64]. There is evidence that etizolam and desmethylclobazam toxicity or side effects are caused by CYP2C19 deficiency [63].

Other drugs tested for the management of chorea in HD include cannabinoids (cannabidiol, nabilone), amantadine and anticonvulsants, such as levetiracetam or topiramate, but available evidence is still very limited [13].

## 4. Pharmacogenetics of the Drugs Used in the Management of Depression, Irritability, Apathy, Anxiety and Psychosis in Huntington’s Disease

According to estimates, between 33 and 76% of HD patients will experience a psychological problem at some point in their lifetimes. These disorders can develop at various stages of the disease course and deteriorate over time as the disease worsens [65,66]. Additionally, it is expected that an astounding 98% of HD patients with motor symptoms will experience at least one psychological symptom or disturbance [67]. Importantly, pre-symptomatic HD gene carriers have a higher prevalence of depression, which can appear up to ten years before motor symptoms [68]. In addition to having a significant negative influence on the patient’s quality of life and autonomy, psychiatric symptoms may also have a major detrimental impact on family members and other caregivers, and can lead to functional impairment [69]. These psychological aspects, rather than the mobility disorder, are thought to be the most devastating to HD patients, and frequently lead to hospitalization and are the best predictors of the requirement for residential care [70]. Therefore, the management of psychiatric symptoms, such as depression, anxiety, and psychosis, is of great relevance for HD patients.

One of the most prevalent psychiatric symptoms in HD is depression, which causes a detrimental effect on the quality of life. As a result, it is important to be proactive in recognizing and treating depression throughout each phase of the disease.

Early detection of mood changes may be possible with psychotherapy and cognitive behavioural therapy. If grade B depression occurs in HD patients [71], an antidepressant may be recommended [12]. A selective serotonin reuptake inhibitor (SSRI) or a serotonin noradrenaline reuptake inhibitor (SNRI) is advised. In the event that sleep is disturbed, mianserin or mirtazapine are other alternatives [12].

CPIC guidelines for SSRI include important information for *CYP2D6* and *CYP2C19* genotypes and dosing of citalopram, escitalopram, fluvoxamine, paroxetine and sertraline [72]. Table 1 summarizes the dosing recommendations for paroxetine and fluvoxamine based on CYP2D6 phenotype, and citalopram, escitalopram and sertraline based on CYP2C19 phenotype [72]. According to our knowledge, there is no current study that evaluates the response to SSRI antidepressants in HD patients, based on their pharmacogenetic features. Further research is warranted.

When the use of a SNRI is preferred, venlafaxine, desvenlafaxine or duloxetine can be used. No clinical guideline recommends including pharmacogenetic information to guide their treatment; however, some important information should be taken into account.

Humans highly metabolize venlafaxine, excreting between 1 and 10% of the administered dose of the unmodified drug in the urine [73]. The main pathway of venlafaxine’s first pass metabolism is demethylation to O-desmethylvenlafaxine [74]. CYP2D6 is the primary enzyme involved in O-desmethylvenlafaxine production [75]. Desvenlafaxine succinate, a salt of O-desmethylvenlafaxine, is an approved drug that is also commercialized. Additionally, CYP3A4 and CYP2C19 catalyse the N-demethylation of venlafaxine to N-desmethylvenlafaxine, which is typically a minor metabolic route [76]. CYP2D6 metabolizer phenotype has been demonstrated to have a clear impact on the pharmacokinetics of venlafaxine, and studies have found a correlation between the CYP2D6 genotype and the metabolic ratio of venlafaxine to O-desmethylvenlafaxine [77,78,79]. The DPWG guideline recommends selecting an alternative to venlafaxine or reducing the dose and monitoring the patient’s plasma metabolite level for patients with CYP2D6 PM and IM phenotypes [36,80]. For CYP2D6 UM, the recommendation is to increase the dose to 150% or select an alternative to venlafaxine [36,80].

There is a need for further research on the combined effect of CYP2D6 and CYP2C19 on venlafaxine metabolism, since it has only been examined in studies with small sample sizes [79,81]. When evaluating pharmacogenomic data for venlafaxine dose modifications, both *CYP2D6* and *CYP2C19* genotypes should be taken into account. This is due to allelic variants in both *CYP2D6* and *CYP2C19* that affect the overall concentration of the active substances venlafaxine and O-desmethylvenlafaxine [79]. Moreover, since CYP2C19 PM and UM phenotypes are present in most populations, it is reasonable to expect that these may have an impact on venlafaxine metabolism, particularly in CYP2D6 PM and IM subjects, in which the formation of O-desmethylvenlafaxine is diminished [74]. In candidate gene association research, the *ABCB1* gene, which codes for the transporter P-glycoprotein, was found to be related to variations in the clinical efficacy of P-glycoprotein substrate antidepressants, like venlafaxine [82].

Only a few candidate gene studies investigate the influence of variants in pharmacodynamic genes such as catechol-O-methyltransferase (*COMT*), serotonin receptor 2A (*HTR2A*), brain-derived neurotrophic factor (*BDNF*), and dopamine transporter (*SLC6A4*), on the variability of treatment outcomes. No significant findings were described for the *COMT* and *BDNF* genes, but an influence of the *HTR2A* and *SLC6A4* genes was observed. Subjects carrying the G allele of the *HTR2A* rs7997012 single nucleotide polymorphism experience superior treatment outcomes over time [83]. Moreover, the dopamine transporter *SLC6A4* variable number of tandem repeats polymorphism influenced the rapid response to antidepressant therapy (SSRI, tricyclics, mirtazapine and venlafaxine) [84]. Unfortunately, a cumulative effect of the variations was not investigated because each separate study only examined one gene. Notably, none of these studies were conducted on HD patients.

Although CYP1A2 and CYP2D6 are mentioned on the FDA label as being involved in duloxetine’s metabolism, there is no particular advice based on these specific enzymes’ metabolic phenotype [85]. Strong CYP2D6 inhibitors may change the concentrations of duloxetine, according to the FDA label, which also advises against using them [85]. Differences in CYP1A2 expression or activity levels may account for higher duloxetine plasma concentrations in non-smokers and in women [86]. Further research is warranted to better establish the influence of *CYP1A2* polymorphisms on enzyme activity, and, therefore, duloxetine efficacy in HD patients.

Mirtazapine is extensively metabolized in the liver by the isoenzymes CYP1A2, CYP2D6 and CYP3A4 [87]. It is helpful for those with depression who also have symptoms of anxiety and sleep disturbances [87]. An impact of *CYP2D6* genotype on steady-state serum concentrations of the enantiomers of mirtazapine and its metabolites was found in the study by Lind et al. [88]. However, the small proportion of CYP2D6 UM and PM found precludes us from drawing firm conclusions. A study performed in 45 patients with major depressive episodes found that in non-smokers, the plasma levels of mirtazapine and its metabolites depended on the *CYP2D6* genotype [89]. Therefore, the influence of the *CYP2D6* genotype appears to be hidden by the elevated CYP1A2 activity observed in smokers [89].

An extremely typical HD symptom is irritability. Impatience and a propensity to become upset at the slightest provocation are the defining traits of this changeable disorder. Impulsivity favours overflow and lack of control, which can result in hostile conduct toward oneself or others and, in exceptional cases, criminal action [12]. This symptom may be brought on by the patient’s annoyance over the significant loss of his abilities, difficulties in expressing himself, neurological/psychological tiredness brought on by the latter, and the patient’s feelings of frustration [12]. Potential environmental factors underlying the patient’s annoyance and irritability should be investigated before beginning pharmaceutical treatment [12]. SSRIs are first-line treatments for irritability, but in order to be successful, they may need to be taken at or close to the maximum dosage [12]. When sleep disturbances are present, combined therapy with mirtazapine may be beneficial for irritable patients who do not respond well to an SSRI alone [12]. A neuroleptic is advised as the first line of treatment for patients who exhibit aggressive conduct [12]. SSRIs and mirtazapine pharmacogenetics have already been discussed. Regarding neuroleptics, one of the most widely used is haloperidol, which has therapy recommendations based on *CYP2D6* genotype, as discussed above.

Levy and Czernecki’s definition of apathy as “a quantitative loss in goal-directed behaviour” [90], states that it shows up clinically as a decline in interest, spontaneity, motivation, and drive. It is the most prevalent psychological and behavioural symptom of HD, particularly in the middle and later stages, and it significantly reduces daily living activities. Apathy and irritation are two sides of the same cognitive and psychological symptom [91]. Apathy may intensify with depression, and a SSRI is then advisable. Moreover, it is recommended to prevent unnecessary prescriptions of sedatives or to limit their dosage, as they may cause apathy. In this regard, pharmacogenetic information might be useful to guide the treatment.

In HD, anxiety—defined as the uneasy sensation of apprehension or worry about something that has happened or might have happened—is prevalent. In addition to being correlated with family, social, and economic problems, as well as the burden of the patient’s disease, anxiety is linked to the other symptoms (motor and cognitive), as the patient is nervous due to the loss of important functions [12]. Anxiety is linked to irritation, diminished quality of life, pain, sickness beliefs, coping, and depression. SRI or SNRI are first-line treatments for anxiety, especially when it coexists with depression. Anxiolytics that are prescribed on demand may be advantageous, but care must be taken because of the potential for falls or exacerbation of existing conditions. When other therapies for anxiety are unsuccessful, neuroleptics are useful therapeutic alternatives [12]. Important pharmacogenetic traits of SSRI, SNRI and neuroleptics have already been mentioned above. Additionally, a benzodiazepine should be prescribed as needed when agitation is linked to an anxiety disorder, to lower the risk of dependence and falls. Although it should be avoided as much as possible, some individuals may nevertheless require long-term benzodiazepine medication. Some pharmacogenetic information regarding the use of benzodiazepines is already depicted in Section 3.4.

When a person becomes detached from reality, they experience psychosis. Hallucinations are described as a perception without an object that the person clings to and responds to as though it originated outside of them. Delusions are erroneous ideas that are founded on faulty assumptions about the surrounding world, as well as the cultural and social environment to which the patient belongs. Hallucinations and delusions are first treated with second-generation neuroleptics [12]. In the case of akinetic types of HD with severe parkinsonian symptoms, clozapine should be recommended as the first-line therapy. The patient may benefit from treatment with serotoninergic antidepressants combined with an atypical neuroleptic in situations when persistent ideation mimics psychotic symptoms. The FDA label raises concerns about CYP2D6 PM and possible drug–drug interactions with drugs metabolized by the same CYP450 enzymes as clozapine (CYP1A2, CYP2D6 and CYP3A4) [92], despite the fact that there are currently no published pharmacogenetic guidelines for patients with variants influencing clozapine metabolism.

## 5. Other Biomarkers in Study

In a previous study, we found a possible association between apathy and impaired social cognition and some polymorphisms in the oxytocin receptor coding gene (*OXTR*) that might be some evidence of hypothalamic degeneration in HD, leading to oxytocinergic dysfunction in HD and its association with neuropsychiatric manifestations [93]. Polymorphisms in the oxytocin receptor could have a relevant role in the correct development of social and cognitive functions. We found various *OXTR*-synonymous variants that warrant further study for their involvement in apathy, irritability, and social cognition in HD [93].

## 6. Future Perspectives

HD remains a difficult-to-treat disease, in need of novel and effective therapies. The long-term goal of pharmacogenetic studies is to use genotype data to predict the effective treatment response to a specific drug and, in turn, prevent potential undesirable effects of its administration. Pharmacogenetic information could be helpful to optimize HD antichoreic, antidepressant and antipsychotic treatment to deal with movement disorders, depression, anxiety and psychosis, whose symptoms place an enormous burden on patients’ quality of life.

Majority of the findings in this analysis came from retrospective observational cohort studies, without any independent verification of these specific conclusions. Many dose-adjustment recommendations come from pharmacokinetic studies and have not shown a direct association with clinical outcomes, which is an important limitation. Additional strategies should surely conduct prospective, randomized clinical trials in larger populations to test predetermined hypotheses. The restrictions of the candidate gene approach, which prevent the detection of causal variants that are not included in the study, are another factor to take into account. In order to direct the development and application of new therapies that improve response to treatment, increase its effectiveness, and reduce toxicity, it is anticipated that the development of genome sequencing techniques will improve access to pharmacogenetic information on patients and diseases in the forthcoming years.

The impact of pharmacogenetic features on the response to antidepressants and antipsychotics is well documented in psychiatric patients, but this approach has not been investigated in patients with HD. However, it is clear that extrapolation of evidence from one group of patients to others is not the appropriate approach considering the specificity of the clinical history, medical condition, or specific treatment, which may influence the therapeutic outcome. Recommendations on the conduct of a pharmacogenetic test prior to therapy are a premature approach that we cannot currently adopt, given the limited level of data. Future research should address these issues to ensure that clinical use is appropriately supported, feasible, and applicable. Therefore, it is imperative to evaluate and confirm all these findings in a very large and well-phenotyped population of HD patients.

It would be beneficial to conduct an in-depth pharmacogenetic study to gauge how patients respond to the main medications described in this review for treating HD. A genome-wide association study (GWAS) involving a large number of patients would be the best course of action, because it may enable the discovery of previously unidentified genes and variants related to drug effects, without the need for an in-depth understanding of the physiology of the disease, or the pharmacokinetics or pharmacodynamics of the drug. However, due to the increased expenses associated with this method compared to the analysis of candidate genes, not all research groups have access to it. In the event that the latter methodology is used, we have listed the most pertinent genetic variants in Table 2 to aid in deciding which ones would be worthwhile to investigate.

Finally, the necessity to identify suicidal ideations appears as a concept to which we must pay particular attention. Suicidal ideation or attempts are frequent in HD and are associated with depression, a history of prior suicide attempts, and family history of suicide [94]. Treating risk factors, such as underlying depression, social isolation, and impulsivity, might help to prevent suicide. Moreover, it would be of great relevance to carry out a pharmacogenetic study to evaluate the number of self-injurious events or suicidal ideations in order to check whether a better pharmacogenetic adjustment of antidepressant and antipsychotic drugs can reduce these indicators.

## Figures and Tables

**Table 1:** Dosing recommendations for paroxetine, fluvoxamine, citalopram, escitalopram and sertraline based on CYP2D6 or CYP2C19 phenotype.

Phenotype *	Paroxetine	Fluvoxamine	Citalopram	Escitalopram	Sertraline
UM	Consider alternative drug not predominantly metabolized by CYP2D6.	No recommendation.	Consider an alternative drug not predominantly metabolized by CYP2C19.	Consider an alternative drug not predominantly metabolized by CYP2C19.	If patient does not respond to recommended starting dose, consider alternative drug not predominantly metabolized by CYP2C19.
NM	Initiate therapy with recommended starting dose.	Initiate therapy with recommended starting dose.	Initiate therapy with recommended starting dose.	Initiate therapy with recommended starting dose.	Initiate therapy with recommended starting dose.
IM	Initiate therapy with recommended starting dose.	Initiate therapy with recommended starting dose.	Initiate therapy with recommended starting dose.	Initiate therapy with recommended starting dose.	Initiate therapy with recommended starting dose.
PM	Consider alternative drug not predominantly metabolized by CYP2D6 or consider a 50% reduction of recommended starting dose.	Consider alternative drug not predominantly metabolized by CYP2D6 or consider a 25–50% reduction of recommended starting dose.	Consider alternative drug not predominantly metabolized by CYP2C19 or consider a 50% reduction of recommended starting dose.	Consider alternative drug not predominantly metabolized by CYP2C19 or consider a 50% reduction of recommended starting dose.	Consider alternative drug not predominantly metabolized by CYP2C19 or consider a 50% reduction of recommended starting dose.
	CYP2D6 *		CYP2C19 *		

* refers to the phenotype of either CYP2D6 or CYP2C19. Abbreviations: CYP2C19: cytochrome P450 family 2 subfamily C member 19; CYP2D6: cytochrome P450 family 2 subfamily D member 6; UM, ultrarapid metabolizer; NM: normal metabolizer; IM: intermediate metabolizer; PM: poor metabolizer. Adapted from: Hicks et al. [72].

**Table 2:** An overview of the relevant gene variations for the drugs addressed in this review.

Gene	Drug(s) Related	HD Symptom
*ABCB1*	Risperidone	Chorea
		Psychiatric symptoms
		Anxiety
	Olanzapine	Chorea
		Psychiatric symptoms
		Anxiety
	Venlafaxine	Depression
		Anxiety
*AKT1*	Risperidone	Chorea
		Psychiatric symptoms
		Anxiety
*ANKK1*	Aripiprazole	Chorea
		Psychiatric symptoms
		Anxiety
*BDNF*	Venlafaxine	Depression
		Anxiety
*CCL2*	Risperidone	Chorea
		Psychiatric symptoms
		Anxiety
*COMT*	Risperidone	Chorea
		Psychiatric symptoms
		Anxiety
	Venlafaxine	Depression
		Anxiety
*CYP1A2*	Olanzapine	Chorea
		Psychiatric symptoms
		Anxiety
	Duloxetine	Depression
		Anxiety
	Clozapine	Chorea
		Psychiatric symptoms
		Anxiety
		Psychosis
	Mirtazapine	Depression
*CYP2C19*	Escitalopram	Depression
		Irritability
		Apathy
		Anxiety
	Citalopram	Depression
		Irritability
		Apathy
		Anxiety
	Sertraline	Depression
		Irritability
		Apathy
		Anxiety
	Venlafaxine	Depression
		Anxiety
	Diazepam	Agitation and anxiety
*CYP2D6*	Tetrabenazine	Chorea
	Deutetrabenazine	
	Aripiprazole	Chorea
		Psychiatric symptoms
		Anxiety
		Psychosis
	Risperidone	Chorea
		Psychiatric symptoms
		Anxiety
		Psychosis
	Olanzapine	Chorea
		Psychiatric symptoms
		Anxiety
		Psychosis
	Clozapine	Chorea
		Psychiatric symptoms
		Anxiety
		Psychosis
	Haloperidol	Chorea
		Psychiatric symptoms
		Irritability
		Anxiety
		Psychosis
	Paroxetine	Depression
		Irritability
		Apathy
		Anxiety
	Fluvoxamine	Depression
		Irritability
		Apathy
		Anxiety
	Venlafaxine	Depression
		Anxiety
	Duloxetine	Depression
		Anxiety
	Mirtazapine	Depression
		Irritability
*CYP3A4*	Aripiprazole	Chorea
		Psychiatric symptoms and anxiety
	Risperidone	Chorea
		Psychiatric symptoms
		Anxiety
	Olanzapine	Chorea
		Psychiatric symptoms
		Anxiety
	Clozapine	Chorea
		Psychiatric symptoms
		Anxiety
		Psychosis
	Haloperidol	Chorea
		Psychiatric symptoms
		Anxiety
	Mirtazapine	Depression
	Diazepam	Agitation and anxiety
	Clobazam	Agitation and anxiety
	Midazolam	Agitation and anxiety
	Clonazepam	Agitation and anxiety
	Alprazolam	Agitation and anxiety
	Tiazolam	Agitation and anxiety
*DAOA*	Aripiprazole	Chorea
		Psychiatric symptoms
		Anxiety
*DRD2*	Aripiprazole	Chorea
		Psychiatric symptoms
		Anxiety
	Olanzapine	Chorea
		Psychiatric symptoms
		Anxiety
*DRD3*	Olanzapine	Chorea
		Psychiatric symptoms
		Anxiety
*HTR2A*	Olanzapine	Chorea
		Psychiatric symptoms
		Anxiety
	Venlafaxine	Depression
		Anxiety
*LEP*	Olanzapine	Chorea
		Psychiatric symptoms
		Anxiety
*OXTR*	No drug—related	Apathy
		Irritability
		Impaired social cognition
*SLC6A4*	Venlafaxine	Depression
		Anxiety
*UGT1A4*	Haloperidol	Chorea
		Psychiatric symptoms
		Anxiety
*UGT1A9*	Haloperidol	Chorea
		Psychiatric symptoms
		Anxiety
*UGT2B7*	Haloperidol	Chorea
		Psychiatric symptoms
		Anxiety

## Data Availability

Not applicable.
